# Feasibility of a Psychoeducational Intervention for Empowering Parents to Optimise Feeding Practices in China: A Randomised Controlled Feasibility Trial

**DOI:** 10.1111/mcn.70155

**Published:** 2026-01-08

**Authors:** Jian Wang, Yan‐Shing Chang, Xiaoxue Wei, Yang Cao, Kirsty Winkley

**Affiliations:** ^1^ Florence Nightingale Faculty of Nursing, Midwifery and Palliative Care King's College London London UK; ^2^ Department of Haematology and Oncology Shanghai Children's Medical Centre Affiliated to Shanghai Jiao Tong University School of Medicine Shanghai China; ^3^ Clinical Epidemiology and Biostatistics, School of Medical Sciences, Faculty of Medicine and Health Örebro University Örebro Sweden; ^4^ Unit of Integrative Epidemiology, Institute of Environmental Medicine Karolinska Institutet Stockholm Sweden

**Keywords:** feeding practices, parents, preschool children, psychoeducational intervention, weight perception

## Abstract

Parental feeding practices play a crucial role in preventing childhood obesity and promoting healthy eating habits. However, few interventions are specifically designed to improve these practices. We aimed to assess the feasibility and preliminary effects of a novel psychoeducational intervention, Empower Parents to Optimise Feeding Practices (EPO‐Feeding), tailored to enhance parental feeding practices in China. A parallel‐arm feasibility randomised controlled trial (RCT) was conducted in two public kindergartens in Yangzhou, China. Participants were randomly assigned to the intervention group (EPO‐Feeding programme plus usual care) or control group (usual care). Data were collected at baseline, post‐intervention, and 1 month after intervention. Descriptive statistics assessed feasibility and acceptability, while analysis of variance for repeated measures and generalised estimating equations analysed continuous and categorical outcomes across time points, respectively. Within 2 weeks, 131 parents expressed interest, and 84 eligible participants were randomly assigned. Module attendance and retention rates were high, with 83.3% (*n* = 35) completing all sessions and 97.6% (*n* = 82) completing all measurements. Satisfaction surveys indicated high acceptability. Statistically significant improvements were observed in the intervention group, including increased encouragement of healthy eating and monitoring, reduced pressure to eat and food as rewards, improved weight accurate perception, and enhanced parenting efficacy (*p* < 0.05). However, no significant effects were found in long‐term outcomes, including children's eating behaviours and weight status. This study demonstrates high feasibility and acceptability of the EPO‐Feeding programme and suggests its potential to support Chinese parents' feeding strategies. A full‐scale RCT is recommended.

**Trial registration**. It was registered with ClinicalTrials.gov (NCT06181773), 20/11/2023.

## Summary

- Parental feeding practices play a critical role in preventing childhood obesity, yet culturally tailored interventions remain scarce in non‐Western contexts (e.g., China).
- Accurate parental perception of child weight status is essential for improving feeding practices.
- This is the first feasibility trial of a psychoeducational programme aimed at improving Chinese parents' feeding practices, showing high feasibility, acceptability, and promising behavioural change outcomes.
- The intervention shows potential for broader implementation across community, clinical, and educational settings.

## 1 Introduction

Childhood overweight and obesity remain pressing concerns worldwide. These conditions often track into adulthood and are associated with an increased likelihood of developing chronic illnesses (Geng et al. 2018). In 2020, an estimated 7% of Chinese children under 6 years old were classified as overweight, with 3.6% identified as obese, positioning China as having the largest population of children with obesity worldwide (Pan et al. 2021). Eating behaviours are well‐recognised determinants of childhood overweight and obesity (Spruijt‐Metz 2011). Understood within a dynamic ecological framework (Dev et al. 2013), parents, often as primary caregivers in the family unit, exert a substantial influence on children's dietary habits and weight outcomes through feeding approaches (Faith et al. 2004; Ruzicka et al. 2021). The preschool years represent a critical window for establishing optimal feeding strategies and promoting long‐term healthy eating habits (Spruijt‐Metz 2011). Thus, research increasingly aims to support parents in improving feeding practices for preschoolers.

Feeding practices refer to practices or strategies that caregivers employ to manage what, when and how much their children eat and to shape their child's eating behaviours (Vaughn et al. 2016; Wang, Zhu, et al. 2022). These practices are generally classified into two main types: non‐responsive feeding (e.g., pressure to eat) and responsive feeding (e.g., monitoring) (Savage et al. 2017; Wang, Zhu, et al. 2022). Non‐responsive feeding is characterised by caregiver‐centred strategies that prioritise caregivers' goals over children's needs (Vaughn et al. 2016; Wang, Zhu, et al. 2022). On the other hand, responsive feeding focuses on encouraging children to eat autonomously based on their physiological and developmental needs (Pérez‐Escamilla et al. 2021; Savage et al. 2017). Non‐responsive feeding is closely related to a high risk of childhood obesity (Ruzicka et al. 2021) and problematic eating behaviours (Wang, Wu, et al. 2023; Wang, Zhu, et al. 2022). However, responsive feeding has been reported to mitigate these risks (Wang, Wu, et al. 2023). Consequently, optimising feeding strategies has become a key target for interventions to promote children's healthy eating and prevent childhood obesity.

A recent systematic review of 18 programmes, which used feeding practices as an outcome, reported inconsistent effects on feeding practices, with numerous studies showing non‐significant outcomes (Wang et al. 2024). This lack of impact may be attributed to the limited number of interventions prioritising feeding practices as a focus (Ledoux et al. 2018). Instead, most interventions primarily addressed broader child nutrition‐related issues, such as healthy eating promotion and obesity prevention (Gomes et al. 2018; Haire‐Joshu et al. 2008; Hart et al. 2016). These findings highlight the absence of standardised components to optimise feeding practices. Thus, it is essential to design interventions that effectively target these practices.

It is well established that psychoeducational programmes provide a structured framework that goes beyond traditional knowledge transfer to promote sustainable behavioural change (Charalampopoulos et al. 2017). Similarly, research indicates that psychoeducational feeding interventions, involving structured sessions, group discussions, and motivational interviewing, predominantly report significant improvements in certain feeding practices (Hammersley et al. 2019; Mobley et al. 2023; Sobko et al. 2020). However, a programme that exclusively focused on educational components using technology‐based self‐directed educational resources demonstrated no significant changes in feeding practices (Duncanson et al. 2016). In the broader context of child health promotion, psychoeducational interventions offer education as a foundation supplemented by psychological elements (e.g., emotional support), which may significantly enhance participants' problem‐solving abilities and coping strategies for managing challenges (Kurnik Mesarič et al. 2024). Thus, the development of psychoeducational interventions is vital to bridge existing gaps and promote effective optimisation of feeding practices.

Furthermore, empirical evidence indicates that supporting the accurate perception of child weight may be a crucial first step toward implementing appropriate feeding practices (Wang, Winkley, et al. 2023; Wang, Zhu, et al. 2022). Although only a few feeding interventions have considered this factor (Flores‐Peña et al. 2022; Gomes et al. 2018), educating parents to accurately perceive and appropriately respond to their child's weight may be crucial for promoting effective feeding practices. For instance, a randomised controlled trial (RCT) in Mexico (*n* = 294) conducted 4‐weekly group sessions (e.g., what is children's weight) primarily aimed to improve mothers' accurate perception of children's weight, which led to a greater improvement in weight perception accuracy and feeding practices (Flores‐Peña et al. 2022). Additionally, feeding practices are deeply influenced by cultural beliefs (Li et al. 2015; Zhou et al. 2015). In China, particularly in low‐ to middle‐income areas, parents often overfeed children or give them excessive amounts of food due to the misconception that a higher body weight signifies better nutrition (Li et al. 2015; Zhou et al. 2015). Chinese parents may often use a favourite food as an expression of love or as a means to regulate children's behaviours (Jiang et al. 2007). The cultural preference for “chubby” children leads Chinese caregivers failing to recognise overweight or obesity as a health concern (Jiang et al. 2007; Zhou et al. 2015).

To address these gaps, we developed a novel psychoeducational programme, the Empower Parents to Optimise Feeding Practices (EPO‐Feeding) programme, to support parents in improving feeding practices and accurate perception of preschool children's weight in China. The primary aim was to test the feasibility and acceptability of this programme. The secondary aim was to evaluate its preliminary effects (early signals of potential benefit) on parental feeding practices, accurate perception of child weight, parenting sense of competence, children's eating behaviours and weight status. Findings from this trial could serve as foundational evidence to support a definitive RCT aimed at evaluating its long‐term effectiveness.

## 2 Method

### 2.1 Research Design

This is a parallel‐arm feasibility RCT. The study protocol has been published (Wang, Cao, et al. 2024). This trial was registered with ClinicalTrials.gov (NCT06181773).

### 2.2 Study Setting and Recruitment

This trial was conducted at two public kindergartens located in Yangzhou, which is considered a typical mid‐income city in Jiangsu Province, Eastern China. Recruitment commenced on 8 December 2023 and concluded on 20 December 2023. Following approval from gatekeepers, recruitment was initiated using take‐home letters and posters. In parent meetings, kindergarten teachers provided participant information sheets and consent forms. They further elaborated on the trial objectives and stressed that participation was completely voluntary. Interested parents reached out to the first author (JW). Once eligibility was confirmed, written informed consent was secured before participation.

Parents were deemed eligible if they fulfilled the following criteria: (a) had primary responsibility for their preschool children's eating and family food environment; (b) had at least one preschooler aged 2–6 years old enrolled in the selected kindergartens. For parents with more than one preschool child, they were advised to focus on the child whose eating habits, weight status, or nutrition raised the most concern; (c) were aged 18 years or older; (d) were able to provide informed consent; (e) were proficient in speaking and writing Chinese. Parents were deemed ineligible if they fulfilled any of the following criteria: (a) diagnosed with severe physical or mental illnesses (e.g., mental retardation) that would prevent participation; (b) having children with medical conditions impacting eating or nutrition (e.g., diagnosed eating disorders); (c) diagnosed with an eating disorder or being pregnant during the duration of the trial; (d) previous participation in interventions related to child growth and nutrition; (e) previous involvement in the EPO‐Feeding programme development.

### 2.3 Sample Size

Sample size determination followed guidelines for the design and evaluation of feasibility pilot studies (Teresi et al. 2022). A sample size of 25 to 50 participants per group is considered appropriate in feasibility trials to assess recruitment and retention rates and to obtain sufficiently precise estimates of variability (e.g., SD) to inform the sample size calculation for a future full‐scale RCT (Teresi et al. 2022; Whitehead et al. 2016). No formal power calculation was undertaken, as the aim of this feasibility trial was not to detect intervention effects. A total of 70 participants is expected to be enrolled in this trial, with an equal allocation of 35 individuals to each group. We expected a 15% attrition rate (Haire‐Joshu et al. 2008; Hart et al. 2016; Sobko et al. 2020), which should ensure at least 60 participants for both arms.

### 2.4 Randomisation Concealment, and Blindness

Following the completion of the baseline assessment, parents were randomly allocated using a concealed computer‐generated randomisation system with an equal 1:1 allocation ratio. The randomisation process was managed by an author (XW) who was not involved in recruiting participants or collecting data. Participants received notification of their group allocation through either SMS or phone call and were provided with a distinct anonymised identifier to ensure confidentiality. Due to the design of the trial, blinding was limited to data collectors and outcome analysts for group assignments. At the follow‐up stage, two kindergarten teachers, blinded to the group allocation, were trained to measure children's height and weight and to distribute follow‐up questionnaires.

### 2.5 Intervention Group

The EPO‐Feeding programme was developed following the Medical Research Council framework (Skivington et al. 2021), drawing on evidence from two systematic reviews (Wang, Chang, et al. 2024; Wang, Winkley, et al. 2023), a cross‐sectional study (*n* = 1779) (Wang et al. 2025), qualitative interviews and focus groups with 68 stakeholders (i.e., parents, teachers and healthcare professionals). Building on these insights, the programme design was guided by the Behaviour Change Wheel (Michie et al. 2011) and Social Cognitive Theory (Bandura 1989). To further refine the programme and trial methods, key stakeholders (i.e., four parents, four healthcare professionals (HCPs), and five kindergarten teachers) were actively engaged prior to the trial implementation.

This evidence‐based, multi‐component intervention targets four core areas: (1) understanding children's eating behaviours, nutritional needs, and growth patterns; (2) keeping a meaningful parent and child role; (3) fostering a healthy and sustainable family food environment; and (4) implementing appropriate feeding practices. Four weekly modules are delivered in face‐to‐face group sessions. This programme comprises HCP‐led sessions, handouts, group discussions, goal setting and review, homework activities, educational videos shared via WeChat group, updates on their child's actual weight, motivational interviewing (individual support), and follow‐up facilitation with weekly infographics. In addition to the baseline (*T*_0_), Intervention group parents received objective feedback on their child's weight status through SMS a few days prior to outcome measurements. The detailed intervention content was reported following the TIDieR checklist (Hoffmann et al. 2014), and further information can be found in the trial protocol (Wang, Cao, et al. 2024).

Parents in the intervention group received the EPO‐Feeding programme plus usual care. The usual care condition involved the distribution of printed dietary guideline materials for child health, as published by the Chinese government and the Nutrition Society. Two HCPs, each with over 10 years of working experience in the child health department of a local hospital, were trained through a series of meetings and discussions to prepare them for programme delivery. Prior to each module, providers presented the content to the first author to maximise delivery fidelity. Each module was delivered twice a week from 26 December 2023 to 17 January 2024 and was scheduled before school pickup, allowing participants to choose a consistent time at their convenience on the kindergarten premises. After each module, participants received a small gift (e.g., storybooks or stickers) as a gesture of appreciation for their time and to promote positive behaviour changes.

### 2.6 Control Group

Participants in the control group were provided with usual care. Following the final data collection, they were provided with all programme materials and a text message regarding their child's actual weight status measured by kindergarten teachers at the final time point (*T*_2_). As an incentive, participants were given access to pre‐recorded modules.

### 2.7 Data Collection

At baseline (*T*_0_), participants completed the questionnaire, including contact information, their children's student number, demographic characteristics and data on secondary outcomes (i.e., feeding practices, perception of child weight, parenting sense of competence, and child eating behaviours). Demographic characteristics were collected at baseline only, including child's characteristics (i.e., sex, age, weight and height, breastfeeding duration), parental characteristics (i.e., age, role, education level, weight, height), and family characteristics (i.e., structure, number of children, and annual income). Feasibility parameters, including the number of parents approached, recruitment and retention rates, session attendance, module completion, homework submission, and follow‐up measurement completion, were recorded by the research team on a standardised tracking sheet and cross‐checked weekly to ensure consistency. Acceptability was assessed post‐intervention (*T*_1_) using an 8‐item 10‐point Likert satisfaction survey, adapted by the research team from satisfaction items commonly used in feasibility trials of feeding interventions (Mobley et al. 2023; Moore et al. 2018) and minimally tailored for the EPO‐feeding programme. In line with feasibility trial methodology (Eldridge et al. 2016), these ratings were summarised descriptively to indicate acceptability rather than to establish psychometric properties. Secondary outcomes were assessed using validated self‐report measures at three time points: baseline (*T*_0_), post‐intervention (*T*_1_), and 1‐month follow‐up (*T*_2_). To minimise the risk of unmasking, two kindergarten teachers who were unaware of group allocation and not involved in other phases. They were trained to distribute and collect questionnaires (including satisfaction survey) and to measure children's weight and height in the kindergarten using student numbers for identification.

### 2.8 Primary Outcomes

In line with the CONSORT (Consolidated Standards of Reporting Trials) extension for pilot and feasibility trials (Eldridge et al. 2016), the primary objectives (i.e., the feasibility and acceptability of the programme) are outlined below:

- Recruitment and retention: (a) the number of eligible participants approached, consented, and randomised; (b) the number who completed the programme; and (c) the number lost to follow‐up (i.e., dropout rate).
- Attendance/Adherence: (a) number of modules attended; (b) number of homework assignments completed.
- Feasibility of measurement tools: (a) the extent of missing data in the questionnaires; (b) completion rates of the questionnaires.
- Acceptability: an anonymous self‐report survey consisted of eight closed questions about participants' satisfaction with this programme (i.e., rating the programme topics, content, format, delivery, and quality). The satisfaction survey employed a 10‐point Likert scale ranging from 1 (extremely dissatisfied) to 10 (extremely satisfied).

### 2.9 Secondary Outcomes

The preliminary effects of the EPO‐Feeding programme were assessed using the following measures:

- **Feeding practices** were measured with the Chinese Preschoolers' Caregivers' Feeding Behaviour Scale (CPCFBS) (Yuan et al. 2019). Five feeding practices were evaluated with this scale: two non‐responsive feeding practices (i.e., pressure to eat and restriction) and three responsive feeding practices (i.e., monitoring, encouragement of healthy eating and modelling). Use of food as a reward, classified as a non‐responsive feeding strategy, was measured through the C‐CFQ (Chinese version of the Child Feeding Questionnaire) (Zheng et al. 2016). Items on both scales were rated using a 5‐point Likert scale. Subscale scores were calculated as the mean of relevant items; higher scores denoted greater adherence to the respective feeding practice. In this study, all CPCFBS subscales demonstrated good internal consistency at each time point (Cronbach's α at *T*_0_ = 0.70–0.88, *T*_1_ = 0.78–0.94 and *T*_2_ = 0.75–0.84). The internal consistency of food as a reward subscale of the C‐CFQ was moderate to good (Cronbach's α at *T*_0_ = 0.64, *T*_1_ = 0.78 and *T*_2_ = 0.61).
- **Perception of preschool child weight.** The C‐CFQ was used to evaluate self‐reported perception of weight (Zheng et al. 2016) using one item, “How would you describe your child's weight?” Responses to this 5‐point Likert scale item were classified into three categories: underweight, normal weight, and overweight/obese. Parental visual perception of child weight was measured using the Parents' Perception of Healthy Weight Scale for children aged 2–6 years (Collins 1991). This scale has been used in empirical studies (Pallan et al. 2011; Wang et al. 2017) on Asian populations. Using a 7‐point sex‐specific visual scale, parents identified the sketch that best matched their child's current body size (1 = severely underweight, 4 = average weight, 7 = obese). Responses were categorised into three groups: ‘thin’, ‘just right’ and ‘heavy’. **Parents' accuracy in perception of child weight** was measured with a misperception versus non‐misperception category, including self‐reported and visual weight misperception. A score of zero indicated non‐misperception, while negative or positive scores indicated misperception.
- **Parenting sense of competence** was assessed with the Chinese version of the Parenting Sense of Competence Scale (Johnston and Mash 1989; Ngai et al. 2007). This scale consisted of two subscales: an 8‐item efficacy subscale assessing perceived parenting competence, and a 9‐item satisfaction subscale measuring satisfaction and comfort in the parenting role (Ngai et al. 2007). A 6‐point Likert scale (from ‘Absolutely disagree’ to ‘Absolutely agree’) was used for all items. Each subscale was calculated by summing item scores, where higher values indicated stronger perceived parenting competence and satisfaction. Both subscales indicated good internal consistency (Cronbach's α at *T*_0_ = 0.79–0.83, *T*_1_ = 0.6–0.86 and *T*_2_ = 0.79–0.81) in this study.
- **Child eating behaviours** were assessed by the Chinese Preschoolers' Eating Behaviour Questionnaire (Jiang et al. 2014). Five common child eating behaviours were assessed: food fussiness, satiety responsiveness, food responsiveness, unhealthy eating habits, and initiative eating. Items were rated on a 5‐point Likert scale, with higher scores indicating greater use of the respective behaviour. Subscale scores were computed by averaging the responses to items within that subscale. In this study, all these included subscales indicated good internal consistency reliability at all time points (Cronbach's α at *T*_0_ = 0.63–0.80, *T*_1_ = 0.65–0.83 and *T*_2_ = 0.65–0.81).
- **Child weight status** was assessed using child age‐standardised body mass index (BMI) *Z*‐scores, calculated following the World Health Organisation (WHO) guidelines, using the software WHO AnthroPlus (v1.0.4, 2009). BMI *Z*‐scores are categorised into four groups: underweight (*Z*‐score < −2), normal weight (−2 ≤ *Z*‐score ≤ 1), overweight (1 < *Z*‐score ≤ 2), and obese (*Z*‐score > 2) (de Onis et al. 2009). Standardised anthropometric equipment was used to measure children's height and weight to the nearest 0.1 cm and 0.1 kg, respectively.

### 2.10 Fidelity

A fidelity checklist (Table S1) was created to evaluate compliance with the intervention protocol, manual, and theoretical frameworks (Borrelli 2011; Borrelli et al. 2005). The assessment was primarily based on audio recordings, observation notes, and qualitative interviews. Each audio recording was randomly selected from each weekly session, which occurred twice a week. A total of four audio recordings were reviewed by two authors (JW and XW) to ensure consistent delivery and implementation. In addition, the first author (JW) attended each module to observe participants' reactions and feedback in real‐time, while making field notes on key aspects of module delivery (e.g., presentation, resource use and interactions). After each module, these field notes were discussed with providers to refine their delivery. A checklist of observation indicators is available in Table S2. Lastly, qualitative data on participants' and providers' experiences with the programme (*n* = 22) were collected and are currently being prepared for publication in a separate paper.

### 2.11 Progress Criteria Assessment

Table S3 shows the pre‐specified criteria used to inform progression to a definitive RCT. The criteria were developed within the research team and included a thorough evaluation of the feasibility and acceptability to confirm if the necessary criteria had been met.

### 2.12 Statistical Analysis

**Figure 1:**
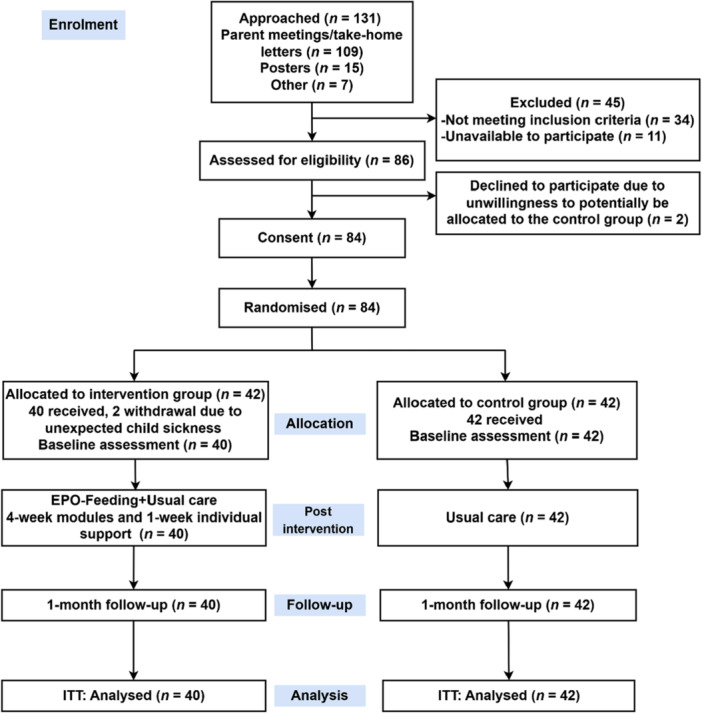
Consolidated Standards of Reporting Trials (CONSORT) flowchart diagram.

Reporting of the feasibility outcomes adhered to the CONSORT guidelines for pilot and feasibility studies (Moher et al. 2001), including a trial flowchart (Figure 1). The primary aim of this feasibility trial was evaluated through descriptive estimates. The criteria for assessing feasibility and acceptability were in line with progress criteria (Table S3). For instance, module retention was defined as at least 80% of participants completing three of the four modules, representing the majority of the intervention content, consistent with prior studies (Gomes et al. 2018; Hammersley et al. 2019).

Exploratory statistical methods were employed to assess the preliminary effects rather than to determine effectiveness. Intention‐to‐treat (ITT) principles were applied by including all participants in the analysis based on their baseline data, with missing data imputed using expectation maximisation. To adhere to ITT principles and conduct a completer analysis, participants were included in the analysis only if they had finished all outcome assessments, attended a minimum of three modules, and had less than 10% missing data at each time point. Cases with over 10% missing data at any time point were removed from the analysis. Baseline comparisons between groups were conducted using independent *t*‐tests and one‐way analysis of variance (ANOVA) for normally distributed continuous variables, and chi‐squared or Fisher's exact tests for categorical variables, with continuity correction applied where appropriate. The expectation maximisation procedure was applied to impute less than 10% missing values, as this technique enhances the power and accuracy of the analysis (Schafer and Graham 2002).

To assess group differences over time, a repeated‐measures ANOVA was conducted with time (*T*_0_, *T*_1_, *T*_2_) as the within‐subjects factor and group allocation as the between‐subjects factor, testing for significant time‐by‐group interactions. Repeated comparisons were corrected using the Bonferroni–Holm procedure. The intervention effects were estimated and reported as effect sizes, using partial eta‐squared (*ηp*²). Effect sizes based on *ηp*² were classified as small (≥ 0.01), medium (≥ 0.06), and large (≥ 0.14) (Cohen 1988). To analyse categorical outcomes across time points, generalised estimating equations were applied (i.e., child weight status and accurate weight perception) between the two groups (Hardin and Hilbe 2008). Outcomes were presented with three major indicators (i.e., group effect, time main effect and group*time effect). The variables that statistically significantly differed between the two groups at baseline were controlled for as confounders in all these models. Data were coded, cleaned, and analysed using SPSS Statistics 29.0 (IBM Corp.). A two‐sided *p*‐value of less than 0.05 was considered statistically significant.

### 2.13 Ethics Statement

Ethical approval for the study was obtained from the Research Ethics Committee at King's College London (HR/DP‐23/24‐39913) and the Institutional Review Board from Baoying Maternal and Child Health Hospital in Yangzhou, China (YZBFYLL‐202303). Written informed consent was obtained from the participants.

## 3 Results

### 3.1 Feasibility: Recruitment

A total of 131 participants were approached within 2 weeks (Table 1). Among these, 86 were assessed as eligible. Around one‐third of potential participants (*n* = 45, 34.4%) were assessed as ineligible (Figure 1). A total of 84 eligible parents agreed to participate, while two did not provide consent due to their unwillingness to be allocated to the control group. A recruitment rate of 64.1% was achieved among individuals who expressed interest in participating. Participants were randomly assigned, with 42 parents allocated to each group. Participant flow is illustrated in the CONSORT diagram provided in Figure 1.

**Table 1:** Recruitment and retention rates and feasibility of measurements.

Variables	Number	
Approach (8 December to 20 December 2023)		
Direct methods: parent meetings, take‐home letters	109	
Indirect methods: poster, consultation	22	
Total approached participants	131	
Ineligible participants	45	
Other caregivers (e.g., grandparents)	18	
Not primary caregivers in the family	4	
Children with conditions affecting eating and nutrition	5	
Participated in the development of intervention	3	
Received similar training	4	
Unavailable to participate (working/routine duty)	11	
Eligible participants	86	
Refused consent	2	
Agreed consent	84	
Randomisation	Intervention (*N*)	Control (*N*)
	42	42
Retention	*N* (mean duration)	
Introduction (26 December/27 December 2023)	40 (26.9 min)	
Session 1 (26 December/27 December 2023)	40 (69.5 min)	
Session 2 (2 January/3 January 2024)	38 (67.6 min)	
Session 3 (9 January/10 January 2024)	37 (76.4 min)	
Session 4 (16 January/17 January 2024)	40 (79.8 min)	
Regular feedback on their questions	40	
Homework activities	After module 1 (40)	
	After module 3 (37)	
	After module 4 (38)	
Regular SMS on their child's actual weight status	40	
Individual support (motivational interviewing via phone call) (17–23 January 2024) by two HCPs	40 (20–30 min)	
Follow‐up weekly key messages of each module via WeChat	40	
**Feasibility of measurements**		
Measurements (Completed)	Intervention (*N*)	Control (*N*)
*T*_0_ (baseline)	40	42
*T*_1_ (24–30 January 2024)	40	42
*T*_2_ (1–7 March 2024)	40	42
Missing data from questionnaires < 10%	Intervention (*N*)	Control (*N*)
*T*_0_	4	3
*T*_1_	1	1
*T*_2_	1	3

### 3.2 Participant Characteristics

The baseline information on this study is presented in Table S4. Most included caregivers (*n* = 77) were mothers and about half of the children (*n* = 39) were boys. Half of the parents had less than a college education. Two‐thirds of families (*n* = 54) had two or more children, and over half (*n* = 47) reported an annual household income below 150,000 Chinese Yuan (CNY) (approximately 0.14 USD per CNY). Additionally, no statistically significant differences were observed between the two groups in participants' baseline characteristics or mean outcome measure scores. Accordingly, no covariates were incorporated into the analysis of preliminary effects (i.e., secondary outcomes).

### 3.3 Feasibility: Retention Rates and Attendance

Out of 84 parents, only two (2.4%) dropped out before providing the baseline assessment, and this was due to their child's sickness. The facilitator (a kindergarten teacher) sent reminder messages via the WeChat group before each module and each follow‐up measurement to promote participants' attendance and completion. Thirty‐five individuals completed all modules, while five individuals attended three modules due to unexpected child sickness (*n* = 3) and work commitments (*n* = 2).

Participation in after‐module homework assignments was not mandatory. After Module 1, an encrypted online survey software (Wenjuanxing) was used to collect parents' assessments of their child's weight and height and estimates of the child's weight status according to distributed handouts (e.g., standards). A total of 40 participants (95.2%) completed this survey. Due to the self‐directed nature of Module 2 homework, completion rates were not monitored. After Modules 3 and 4, we utilised the ‘Jielong’ feature in the WeChat mini programme, which allowed all participants to view and share posts, with 37 (88.1%) participants in Module 3 and 38 (90.5%) in Module 4.

Apart from two participants who withdrew from the intervention group, the remaining participants (*n* = 82, 97.6%) completed the baseline and follow‐up questionnaires. A total of 71 individuals (84.5%) completed all measurements without any missing data, while 11 individuals had less than 10% missing data in their responses.

### 3.4 Acceptability

The satisfaction survey evaluated five elements using eight closed questions rated on a ten‐point scale. High satisfaction scores (mean ± standard deviation [SD]) were recorded for each of these programme elements, specifically topics/themes (9.98 ± 0.15), content (9.78 ± 0.53), format (9.86 ± 0.41), provider delivery (9.98 ± 0.15), and quality (9.88 ± 0.39). Overall, participants gave the EPO‐Feeding programme an overall average rating of 9.87 (SD = 0.25).

### 3.5 Fidelity Assessment

Fidelity assessment of the EPO‐Feeding programme encompassed five core domains: study design, provider training, delivery, receipt, and enactment. Overall, the programme demonstrated high fidelity, with details for each domain presented in Table S5.

### 3.6 Progress Criteria

Table S3 shows the findings for each progression criterion, all of which were met, thereby supporting the progression to a definitive RCT.

### 3.7 Preliminary Effects of the Intervention Compared to the Control Group

Consistent with the feasibility design of this trial, the following results are presented as exploratory estimates of change rather than confirmatory tests of effectiveness. Table 2 reports the mean ± SD scores for participants who completed the study in both groups at baseline and follow‐up assessments. Changes in mean outcomes from baseline were analysed using repeated‐measures ANOVA with pairwise comparisons for both groups (Table S6).

**Table 2:** Preliminary effects of the EPO‐Feeding Programme.

Variables	Group	*T*_0_	*T*_1_	*T*_2_	*F* time	*p*	*ηp* ^2^	*F* group	*p*	*ηp* ^2^	*F* group* time	*p*	*ηp* ^2^
Encouragement of healthy eating	EPO‐Feeding + usual care	4.18 ± 0.52	4.38 ± 0.50	4.38 ± 0.47	0.740	0.479	0.009	1.956	0.166	0.024	4.177*	0.017	0.050
	Usual care	4.24 ± 0.50	4.18 ± 0.58	4.12 ± 0.58									
Modelling	EPO‐Feeding + usual care	3.98 ± 0.78	4.33 ± 0.57	4.18 ± 0.65	2.153	0.119	0.026	0.715	0.400	0.009	1.576	0.210	0.019
	Usual care	4.02 ± 0.97	4.04 ± 0.93	4.08 ± 0.77									
Monitoring	EPO‐Feeding + usual care	4.06 ± 0.75	4.18 ± 0.83	4.41 ± 0.42	0.947	0.390	0.012	0.267	0.607	0.003	5.983**	0.003	0.070
	Usual care	4.18 ± 0.81	4.21 ± 0.81	4.04 ± 0.67									
Pressure to eat	EPO‐Feeding + usual care	3.18 ± 0.86	2.84 ± 0.94	2.53 ± 0.63	9.192***	0.001	0.103	10.472**	0.002	0.116	3.295*	0.040	0.040
	Usual care	3.45 ± 0.85	3.27 ± 0.93	3.29 ± 0.84									
Food as a reward	EPO‐Feeding + usual care	3.45 ± 0.74	2.98 ± 0.63	2.80 ± 0.67	9.336***	0.001	0.105	13.299***	0.001	0.143	4.581*	0.012	0.054
	Usual care	3.63 ± 0.89	3.56 ± 0.93	3.51 ± 0.74									
Restriction	EPO‐Feeding + usual care	3.49 ± 0.84	3.79 ± 0.78	3.59 ± 0.64	3.969*	0.021	0.047	0.034	0.854	0.000	1.416	0.246	0.017
	Usual care	3.43 ± 0.97	3.63 ± 0.88	3.73 ± 0.87									
Food fussiness	EPO‐Feeding + usual care	2.99 ± 0.78	2.80 ± 0.72	2.96 ± 0.73	1.110	0.332	0.014	0.000	0.996	0.000	2.555	0.081	0.031
	Usual care	2.95 ± 0.74	2.96 ± 0.70	2.84 ± 0.65									
Satiety responsiveness	EPO‐Feeding + usual care	2.62 ± 0.74	2.55 ± 0.62	2.61 ± 0.65	0.835	0.436	0.010	0.000	0.983	0.000	0.117	0.890	0.001
	Usual care	2.59 ± 0.68	2.56 ± 0.57	2.61 ± 0.66									
Food responsiveness	EPO‐Feeding + usual care	2.58 ± 0.65	2.48 ± 0.55	2.48 ± 0.56	0.259	0.772	0.003	0.963	0.329	0.012	0.924	0.399	0.011
	Usual care	2.38 ± 0.61	2.41 ± 0.53	2.42 ± 0.53									
Emotional eating	EPO‐Feeding + usual care	1.67 ± 0.53	1.65 ± 0.65	1.70 ± 0.61	1.649	0.196	0.020	1.504	0.224	0.018	0.945	0.391	0.012
	Usual care	1.70 ± 0.54	1.80 ± 0.54	1.90 ± 0.55									
Initiative eating	EPO‐Feeding + usual care	4.00 ± 0.53	4.00 ± 0.44	3.89 ± 0.51	0.727	0.485	0.009	2.799	0.098	0.034	1.417	0.245	0.017
	Usual care	3.79 ± 0.60	3.78 ± 0.53	3.80 ± 0.52									
PSOC: efficacy^b^	EPO‐Feeding + usual care	37.38 ± 4.82	39.08 ± 3.62	39.30 ± 3.14	2.263	0.114	0.028	4.050*	0.048	0.048	3.472*	0.039	0.042
	Usual care	36.90 ± 4.88	37.05 ± 5.56	36.45 ± 5.38									
PSOC: satisfaction	EPO‐Feeding + usual care	35.03 ± 8.75	36.00 ± 9.45	37.35 ± 8.03	3.203	0.043	0.038	0.199	0.656	0.002	2.006	0.138	0.024
	Usual care	36.21 ± 7.61	37.64 ± 6.25	36.71 ± 8.00									

*Note:* Data are shown as mean ± SD.

The statistical test used is repeated‐measure analysis of variance. Adjustment for multiple comparisons: Bonferroni.

All models tested Box's Test of Equality of Covariance Matrices and Levene's Test of Equality of Error Variances.

Based on the results of Mauchly's Test of Sphericity, tests of within‐subjects effects with Greenhouse‐Geisser results^b^ (Mauchly's Test *p* < 0.05) and Sphericity Assumed (Mauchly's Test *p* > 0.05) were selected.

Abbreviation: PSOC, Parenting Sense of Competence Scale.

* *p* < 0.05

** *p* < 0.01

*** *p* < 0.001.

Regarding the statistically significant main effects of time, a medium effect size was found for the use of food as a reward (*F*_(2,160)_ = 9.336, *p* < 0.001, *ηp*² = 0.105) and pressure to eat (*F*_(2,160)_ = 9.192, *p* < 0.001, *ηp*² = 0.103). A small effect size was found for restriction (*F*_(2,160)_ = 3.969, *p* = 0.021, *ηp*² = 0.047). The results indicated that the mean scores of these outcome variables changed significantly across three time points. We also found three statistically significant main effects of the group with a medium effect size: pressure to eat (*F*_(1,80)_ = 10.472, *p* = 0.002, *ηp*² = 0.116), use of food as a reward (*F*_(1,80)_ = 13.299, *p* < 0.001, *ηp*² = 0.143), and parenting sense of efficacy (*F*_(1,80)_ = 4.050 *p* = 0.048, *ηp*² = 0.048). The findings indicated statistically significant differences in mean scores between the two groups. Furthermore, five outcomes showed statistically significant group × time interaction effects, suggesting that mean score trajectories differed between the two groups.

#### 3.7.1 Preliminary Effects on Three Responsive Feeding Practices

**Figure 2:**
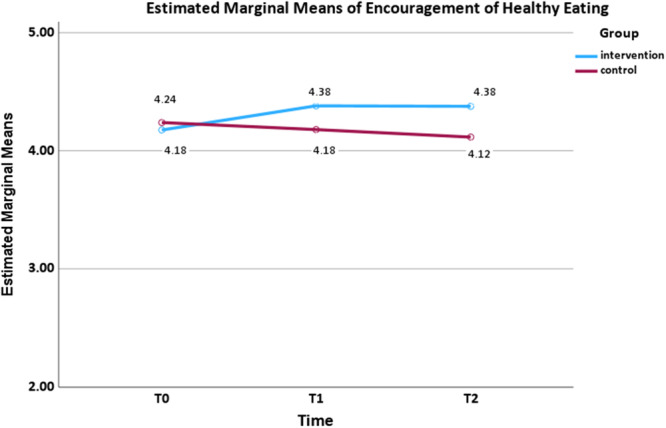
Interaction effect for mean encouragement of healthy eating scores from baseline to follow‐ups by two arms.

**Figure 3:**
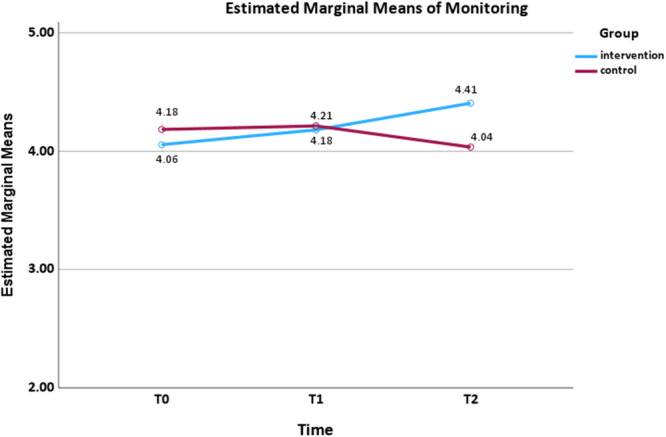
Interaction effect for mean monitoring scores from baseline to follow‐ups by two arms.

Regarding parental encouragement of healthy eating (Figure 2), a small interaction effect size was found (*F*_(2,160)_ = 4.177, *p* = 0.017, *ηp*² = 0.050). The mean scores of the encouragement of healthy eating in the intervention group increased sharply at *T*_1_ (mean difference = −0.204, *p* = 0.013, 95% confidence interval [CI]: −0.373, −0.035) and then remained approximately stable at *T*_2_ (mean difference = −0.200, *p* = 0.061, 95% CI: −0.407, 0.007), whereas the control group showed a decline over time. Regarding monitoring (Figure 3), a medium interaction effect size was observed (*F*_(2,160)_ = 5.983, *p* = 0.003, *ηp*² = 0.070). Monitoring in the intervention group increased over time at *T*_1_ (mean difference = −0.125, *p* = 0.554, 95% CI: −0.357, 0.107) and *T*_2_ (mean difference = −0.350, *p* = 0.010, 95% CI: −0.629, −0.071). In contrast, the control group showed a slight increase followed by a sharp decline. No statistically significant group × time interaction effects were observed for modelling.

#### 3.7.2 Preliminary Effects on Three Non‐Responsive Feeding Practices

**Figure 4:**
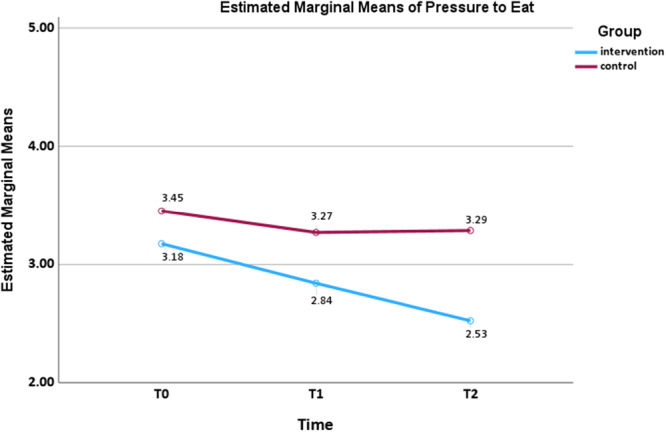
Interaction effect for mean pressure to eat scores from baseline to follow‐ups by two arms.

**Figure 5:**
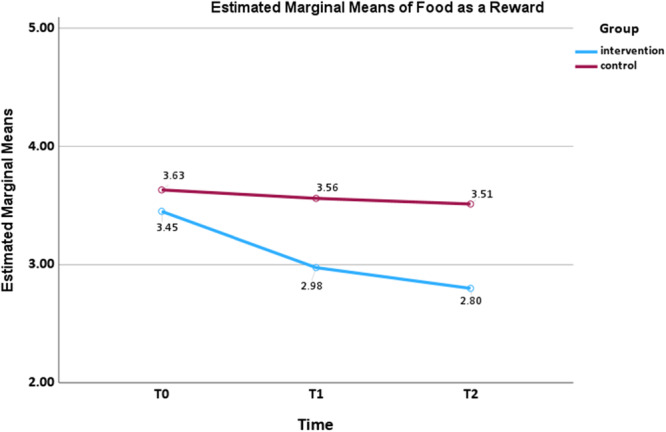
Interaction effect for mean food as a reward scores from baseline to follow‐ups by two arms.

A small interaction effect size was found for pressure to eat (*F*_(2,160)_ = 3.295, *p* = 0.040, *ηp*² = 0.040) and use of food as a reward (*F*_(2,160)_ = 4.581, *p* = 0.012, *ηp*² = 0.054), as shown in Figures 4 and 5, respectively. The mean scores of pressure to eat in the intervention group decreased over time at *T*_1_ (mean difference = 0.333, *p* = 0.042, 95% CI: 0.009, 0.658) and *T*_2_ (mean difference = 0.650, *p* < 0.001, 95% CI: 0.244, 1.056), while the control group showed an initial decrease, followed by a modest increase. Use of food as a reward in the intervention group showed a continuous decline at *T*_1_ (mean difference = 0.475, *p* = 0.001, 95% CI: 0.165, 0.785) and *T*_2_ (mean difference = 0.650, *p* < 0.001, 95% CI: 0.333, 0.967), whereas the control group showed a slight decrease over time. No statistically significant group × time interaction effects were observed for restriction.

#### 3.7.3 Preliminary Effects on Parenting Sense of Competence and Child Eating Behaviours

**Figure 6:**
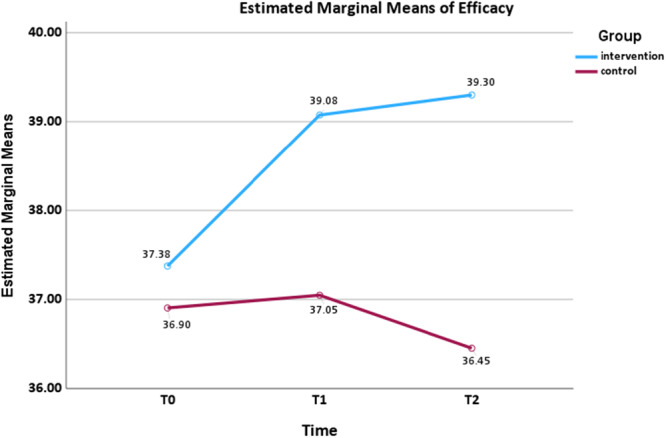
Interaction effect for the sum efficacy scores from baseline to follow‐ups by two arms.

Regarding parenting sense of efficacy (Figure 6), a statistically significant interaction effect size was found (*F*_(1.77,141.82)_ = 3.472, *p* = 0.039, *ηp*² = 0.042). Parenting sense of efficacy in the intervention group increased over time at *T*_1_ (mean difference = −1.700, *p* = 0.065, 95% CI: −3.476, 0.076) and *T*_2_ (mean difference = −1.925, *p* = 0.002, 95% CI: −3.237, −0.613), whereas the control group initially showed a slight increase, followed by a sharp decline. No statistically significant group × time interaction effects were observed for parenting sense of satisfaction and child eating behaviours.

#### 3.7.4 Preliminary Effects on Weight Perception Accuracy and Child Weight Status

Generalised estimating equations were applied to compare categorical outcome measures across three time points (Table S7). A statistically significant interaction effect for accurate self‐reported perception of child weight was detected (*p* = 0.038), indicating that the percentage of accurate self‐reported perception in the two groups changed differently over time. However, the interactions for accurate visual perception and actual child weight status were non‐significant over time. We further employed generalised estimating equations to analyse data from the two groups, respectively (Table S8). The results indicated that, over time, intervention group parents reported significantly greater accuracy in perceiving their child's weight than those in the control group at both baseline and follow‐up.

## 4 Discussion

This study tested the feasibility, acceptability, and preliminary effects of a novel psychoeducational EPO‐Feeding programme for Chinese parents of preschool children. Based on pre‐specified progress criteria, this trial reported sufficient recruitment, measurement completion, and retention rates to support a full‐scale RCT. The secondary outcomes suggest that this programme has the potential to empower Chinese parents to improve their feeding practices, support an accurate perception of their child's weight, and increase confidence and knowledge in effective parenting strategies.

Based on recruitment and retention data, module and measurement completion rates, and participants' self‐reported satisfaction by survey, the intervention was deemed feasible and acceptable. First, the rapid recruitment completed within 2 weeks and surpassing the target sample size suggests high levels of interest and engagement among Chinese parents. This efficient process was largely facilitated by the involvement of kindergarten teachers and hospital staff with expertise in child health, which appeared to enhance parents' trust and interest. Some trials aimed at preventing childhood obesity and promoting children's healthy eating habits have used similar methods for efficient recruitment (Gomes et al. 2018; Mobley et al. 2023). These recruitment strategies appear scalable to a full RCT. However, as approximately one‐third of interested individuals were ineligible, future trials should clarify eligibility criteria and strengthen communication about target participant profiles to optimise recruitment efficiency.

Second, high retention rates were reported for the EPO‐Feeding programme. Specifically, 83.3% of parents completed all 4 weekly sessions. In contrast, some intervention studies that utilised feeding practices as an outcome reported relatively low retention rates for session attendance (Engels et al. 2022; Gomes et al. 2018; Mobley et al. 2023). For instance, an online habit‐based intervention programme targeting parental feeding practices, which comprised 6 weekly modules, reported that only 14.7% of participants were categorised as ‘completers’ for having finished at least five modules (Engels et al. 2022). One possible explanation for the high attrition rate is that participants perceived the programme as overly time‐consuming, with most dropouts occurring after 2–3 weeks. As reported in a previous qualitative synthesis (Mytton et al. 2014), appropriate timing and frequency of sessions are key to parental engagement. The high retention observed in this trial suggests that the session length and delivery structure were appropriate and well accepted. For a future full‐scale RCT, maintaining this delivery format while incorporating flexible scheduling options may further enhance participant adherence and reduce attrition. Furthermore, almost all participants in this feasibility study (*n* = 82, 97.6%) completed baseline and follow‐up questionnaires with less than 10% missing data at each time point. These results indicate that the assessments used for evaluation were feasible and well‐accepted, contrasting with high attrition rates for follow‐up questionnaires in other intervention studies (Engels et al. 2022; Mobley et al. 2023). The high completion rate in this trial may have been due to the involvement of teachers in data collection, and the use of reminder messages. For a future full‐scale RCT, maintaining these engagement strategies and embedding automated follow‐up reminders may help to sustain data completeness over longer follow‐up periods.

Third, participants expressed high levels of satisfaction with the programme's topics, content, format, provider delivery, and quality. A few feasibility trials, which focused on improving feeding practices, have also used similar tools to assess participants' satisfaction and consistently reported high levels of satisfaction (Moore et al. 2018). A potential explanation for high satisfaction levels is that such programmes were designed based on participants' needs and offered practical and relevant strategies (e.g., personalised support) that addressed participants' perceived challenges (Moore et al. 2018). Besides, the fidelity of implementation was high, as most sessions closely adhered to prescribed content and structure. Nevertheless, minimal deviations were noted (e.g., limited class interactions), indicating that for a full‐scale RCT, further refinements such as enhancing healthcare provider training in advanced teaching skills, including managing group dynamics and discussions, may be beneficial. When evaluated against the predefined progress criteria, the programme met each criterion for ‘go‐proceed with RCT’, indicating that the programme is feasible for scaling to a full‐scale trial.

Given that the primary focus of the programme was on promoting an accurate perception of children's weight (Module 1), feeding practices (Modules 2 and 4), and parenting strategies and roles (Modules 2 and 3), the observed directional shifts in these outcomes are encouraging. While interpretation remains cautious due to the feasibility design, the pattern and magnitude of change suggest potential for positive behavioural impact. Notably, four of six targeted feeding practices changed in the desired direction. These results aligned with previous intervention studies designed to improve feeding practices (Agras et al. 2012; Ledoux et al. 2018; Mobley et al. 2023), which reported increases in certain responsive feeding and decreases in non‐responsive feeding. A key aspect of these interventions was the emphasis on direct education and skill‐building to improve feeding practices, such as adopting appropriate feeding strategies (Ledoux et al. 2018; Mobley et al. 2023), keeping meaningful feeding roles (Agras et al. 2012), and creating a positive mealtime environment (Ledoux et al. 2018; Mobley et al. 2023). Furthermore, interventions with multiple components and adequate content appear to have a greater likelihood of promoting feeding practices (Hammersley et al. 2019; Mobley et al. 2023; Sobko et al. 2020). Our programme was a psychoeducational intervention that integrated educational and psychological elements to enhance its potential effects.

Notably, no clear between‐group differences were observed in modelling and restriction feeding practices, visual perception of children's weight, or parenting satisfaction. This may be partly attributable to the relatively favourable baseline profiles in these domains, leaving limited room for observable change within a feasibility sample. For example, the mean scores for modelling in two groups were around four out of five on the rating scale, indicating that participants may have already adopted this behaviour, which may have limited the potential improvement. Although the intervention addressed the limitations of visual weight judgement by educating parents about perceptual inaccuracy and signs of abnormal weight, no culturally adapted silhouette chart for Chinese children is currently available, which may have limited the potential programme's impact on visual perception. Nevertheless, an intervention in the UK that used silhouette charts based on UK90 criteria failed to significantly improve accurate visual perception of child weight (Jones et al. 2023). Due to limited evidence, further studies are warranted to evaluate its effectiveness. Additionally, no clear between‐group differences were observed in children's eating behaviours or weight status. Given only 1‐month follow‐up, this trial may not have captured potential longer‐term impacts. Thus, future evaluations are recommended to incorporate longer‐term follow‐ups at 3‐ and 6‐month intervals (Wang, Chang, et al. 2024).

This study has several limitations. First, the reliance on self‐report data may introduce recall biases. Second, although the sample included a sufficient number of Chinese parents with diverse backgrounds (e.g., education level), the majority of participants were mothers. Thus, generalisability to other demographic groups (e.g., fathers or diverse ethnicities) cannot be assumed. Another key limitation is the absence of qualitative data on participants' and providers' experiences with this programme, which would have provided deeper insights. These findings are being prepared for publication in a separate paper. Furthermore, in line with the feasibility nature of the trial, all outcome analyses were exploratory, and the study was not aimed to detect effectiveness. Therefore, observed between‐group differences should be interpreted as preliminary signals rather than confirmatory evidence of impact. Lastly, while the 1‐month follow‐up results were promising, no assessments were conducted to determine if positive changes are maintained in the longer term.

## 5 Conclusion

This is the first feasibility RCT in China of a programme designed to optimise Chinese parents' feeding practices and improve the accuracy of their perception of child weight. The findings demonstrate that the programme is both feasible and acceptable. With minor refinements, such as clarifying eligibility criteria and strengthening facilitator training, the intervention appears ready to progress to a full‐scale RCT. This programme also shows potential to inform public health strategies by providing a structured, evidence‐based approach for supporting parents in promoting feeding strategies. A larger definitive trial with extended follow‐up is warranted to assess longer‐term outcomes, cost‐effectiveness, and pathways for integration into paediatric care and school‐based health initiatives.

## Author Contributions

Jian Wang led the conceptualisation and project administration, performed the research and formal analysis, managed data, resources, software and visualisations, and wrote and refined the original draft. Jian Wang and Xiaoxue Wei contributed to data investigation and curation. Jian Wang, Kirsty Winkley, Yan‐Shing Chang and Yang Cao designed the study. Yan‐Shing Chang, Yang Cao, and Kirsty Winkley supervised the research and revised the manuscript. All authors reviewed and approved the final manuscript.

## Conflicts of Interest

The authors declare no conflicts of interest.

## Supporting information


**Table S1:** Fidelity Checklist for EPO‐Feeding Programme. **Table S2:** Observation checklist of EPO‐Feeding programme. **Table S3:** Progression Criteria for the EPO‐Feeding Programme: A Feasibility Randomised Controlled Trial (RCT). **Table S4:** Baseline characteristics of participants in two groups. **Table S5:** Fidelity assessment of the EPO‐Feeding Programme. **Table S6:** Outcomes comparison for both groups compared to baseline (T_0_). **Table S7:** Generalised estimating equations results for the main effects and interaction effects. **Table S8:** Results of Generalised estimating equation analysis for comparison of outcome.

## Data Availability

The data that support the findings of this study are available on request from the corresponding author. The data are not publicly available due to privacy or ethical restrictions.
