# Supplementary material for: Feasibility of a Psychoeducational Intervention for Empowering Parents to Optimise Feeding Practices in China: A Randomised Controlled Feasibility Trial
Source: Matern Child Nutr. 2026 Jan 8;22(1):e70155. doi: 10.1111/mcn.70155 (PMC12780882; doi:10.1111/mcn.70155)
Supplement: Supplementary file 1 — Table S1: Fidelity Checklist for EPO‐Feeding Programme. Table S2: Observation checklist of EPO‐Feeding programme. Table S3: Progression Criteria for the EPO‐Feeding Programme: A Feasibility Randomised Controlled Trial (RCT). Table S4: Baseline characteristics of participants in two groups. Table S5: Fidelity assessment of the EPO‐Feeding Programme. Table S6: Outcomes comparison for both groups compared to baseline (T0). Table S7: Generalised estimating equations results for the main effects and interaction effects. Table S8: Results of Generalised estimating equation analysis for comparison of outcome. [file MCN-22-e70155-s001.docx]

**Title: Feasibility of a Psychoeducational Intervention for Empowering Parents to Optimise Feeding Practices in China: A Randomised Controlled Feasibility Trial**

**Table S1. Fidelity Checklist for EPO-Feeding Programme**

| **Fidelity domain** | **Fidelity component** | **Please tick** | | | **Assessment method/details** | **Reason for not done or done to some extent** | **When** |
| --- | --- | --- | --- | --- | --- | --- | --- |
|  |  | **Done** | **To some extent** | **Not done** |  |  |  |
| **Study Design** | 1. Provided information about treatment dose in the intervention condition: | | | | - Protocol - Intervention Programme Manual |  | Before trial implementation |
|  | - Length of modules (each session lasts around 60 minutes; individual support with phone calls lasts about 30 minutes per parent) |  |  |  |  |  |  |
|  | - Number of modules (4 modules) |  |  |  |  |  |  |
|  | - Content of training modules |  |  |  |  |  |  |
|  | - Duration of the module over time |  |  |  |  |  |  |
|  | 1. Provided information about treatment dose in the comparison condition | | | | - Protocol - Intervention Programme Manual |  |  |
|  | - Length of training |  |  |  |  |  |  |
|  | - Number of trainings |  |  |  |  |  |  |
|  | - Content of treatment/training |  |  |  |  |  |  |
|  | - Duration of training over time |  |  |  |  |  |  |
|  | 1. Specification of provider credentials that are needed (HCPs) |  |  |  | - Protocol |  |  |
|  | 1. The theoretical model upon which the intervention is based is clearly articulated | | | | - Protocol - Intervention Programme Manual |  |  |
|  | - The active ingredients are specified and incorporated into the intervention |  |  |  |  |  |  |
|  | - Use of experts or protocol review group to determine whether the intervention protocol reflects the underlying theoretical model or clinical guidelines |  |  |  |  |  |  |
|  | - Plan to ensure that the measures reflect the hypothesized theoretical constructs/mechanisms of action |  |  |  |  |  |  |
|  | 1. Potential confounders that limit the ability to make conclusions at the end of the trial are identified |  |  |  | - Protocol |  |  |
|  | 1. Plan to address possible setbacks in implementation (i.e., backup systems or providers) |  |  |  | - Protocol |  |  |
| **Training of Providers** | 1. Description of how providers will be trained (manual of training procedures) |  |  |  | - Protocol |  | Before/during trial implementation |
|  | 1. Standardization of provider training (especially if multiple waves of training are needed for multiple groups of providers) |  |  |  | - Protocol - Regular discussions/training |  |  |
|  | 1. Assessment of provider skill acquisition |  |  |  | - Protocol - Pre-recorded sessions |  |  |
|  | 1. Assessment and monitoring of provider skill maintenance over time |  |  |  | - Observation of sessions/field notes - Discussion after each module |  |  |
|  | 1. Characteristics being sought in a treatment provider are articulated a priori. Characteristics that should be avoided in a treatment provider are articulated a priori |  |  |  | - Protocol: reported characteristics of experiences, and knowledge in the area |  |  |
|  | 1. At the hiring stage, assessment of whether or not there is a good fit between the provider and the intervention (e.g., ensure that providers find the intervention acceptable, credible and potentially efficacious) |  |  |  | - Scheduled discussions |  |  |
|  | 1. There is a training plan that takes into account trainees’ different education experiences and learning styles |  |  |  | - Providers are the HCPs in the relevant fields (protocol) |  |  |
| **Treatment Delivery** | 1. Method to ensure that the content of the intervention is delivered as specified |  |  |  | - Intervention manual - Prepared materials: slides, handouts, videos etc - Observation of sessions - Regular discussions after each session |  | During trial implementation (Observation) |
|  | 1. Method to ensure that the dose of the intervention is delivered as specified |  |  |  | - Intervention manual - Observation of sessions - Regular discussions - Prepared materials |  | During trial implementation (Observation) |
|  | 1. Mechanism to assess if the provider adhered to the intervention plan or in the case of computer-delivered interventions, method to assess participants’ contact with the information* |  |  |  | - Audio recordings of sessions - Observation of sessions /field notes |  | After completing all modules |
|  | 1. Assessment of nonspecific treatment effects |  |  |  | - Audio recordings of sessions - Observation of sessions /field notes - Participants’ acceptability survey |  | After completing all modules |
|  | 1. Used treatment manual |  |  |  | - Intervention manual - Protocol - Prepared materials |  | After completing all modules |
|  | 1. There is a plan for the assessment of whether or not the active ingredients were delivered* |  |  |  | - Audio recordings of sessions - Observation of sessions /field notes |  | After completing all modules |
|  | 1. There is a plan for the assessment of whether or not proscribed components were delivered (e.g., components that are unnecessary or unhelpful) * |  |  |  | - Audio recordings of sessions - Observation of sessions /field notes |  | After completing all modules |
|  | 1. There is a plan for how contamination between conditions will be prevented |  |  |  | - Protocol |  | Before the implementation |
| **Treatment Receipt** | 1. There is an assessment of the degree to which participants understood the intervention |  |  |  | - Participants’ acceptability survey - Interviews |  | After completing all modules |
|  | 1. There is specification of strategies that will be used to improve participant comprehension of the intervention |  |  |  | - Audio recordings: Intervention content from the intervention manual (e.g., goal setting and feedback) |  | After completing all modules |
|  | 1. The participants’ ability to perform the intervention skills will be assessed during the intervention period |  |  |  | - Process evaluation: interviews - Feedback from participants via WeChat or in person |  | After completing all modules |
|  | 1. A strategy will be used to improve the subject performance of intervention skills during the intervention period |  |  |  | - Individual support: motivational interviewing - Providers monitor the WeChat group and regular feedback |  | During trial implementation |
|  | 1. Multicultural factors considered in the development and delivery of the intervention (e.g., provided in native language; protocol is consistent with the values of the target group) |  |  |  | - Intervention development: interviews/focus groups with stakeholders; cross-sectional study - Prepared materials: handouts/key messages |  | Before and during trial implementation |
| **Treatment Enactment** | 1. Participant performance of the intervention skills will be assessed in settings in which the intervention might be applied |  |  |  | - Follow-up assessment (questionnaires) - Process evaluation: semi-structured interviews |  | During and after trial implementation |
|  | 1. A strategy will be used to improve the performance of the intervention skills in settings in which the intervention might be applied |  |  |  | - Individual support: motivational interviewing with phone/WeChat calls - Follow-up key messages - WeChat group for peer support monitored by HCPs - Homework assignments (e.g., self-monitoring checklists for feeding practices) |  |  |

**Table S2. Observation checklist of EPO-Feeding programme**

| To what standard is the core content being delivered? |
| --- |
| Are the standard manual and resources being used? |
| To what extent are the key learning outcomes of each module achieved? |
| Rate the healthcare professionals |
| 1) A variety of approaches to each module using facilitation, presentation, group discussion, homework activities, feedback |
| 2) Engagement/rapport with group |
| 3) Group management/involvement |
| 4) Challenge negative behaviour within group |
| 5) Positively addressed resistance to programme |
| Additional comments: |

**Table S3. Progression Criteria for the EPO-Feeding Programme: A Feasibility Randomised Controlled Trial (RCT)**

| **Elements** | **Go-proceed with RCT** | **Amend-proceed with changes** | **Stop - do not proceed unless changes are possible** | **Reference** | **Meet which criterion?** |
| --- | --- | --- | --- | --- | --- |
| **Feasibility of participant recruitment**  Can 25-50 eligible participants per group be recruited within a 2-3 week timeframe? | If ≥ 35 eligible participants per group | If 25-35 eligible participants per group | If < 25 participants per group | A sample size of 25 to 50 is suitable for assessing feasibility, accurately estimating retention rate differences, and achieving effect sizes between 0.15 and 0.3 (1, 2). | Go-proceed with RCT |
| **Feasibility of participant retention**  Can less than 20% of recruited participants, including follow-up assessment, drop out? | If < 20% of participants drop out | If 20% - 30% of participants drop out | If > 30% of participants drop out | Previous estimations of a dropout rate range from 8% to 22 % in similar RCTs (3-5). | Go-proceed with RCT |
| **Feasibility of module retention**  Can at least 80% of participants complete three out of four modules? | If ≥ 80% of participants complete all the modules | If ≥ 80% of participants complete 3/4 modules | If < 80% of participants complete 3/4 modules | A minimum of 80% of enrolled participants complete three out of four modules (> 80% intervention content) as previous relevant studies did (6, 7). | Go-proceed with RCT |
| **Acceptability of intervention programme**  Can the mean scores of the acceptability survey (range 1-10) be over 6? | If the mean scores of acceptability ≥ 8 | If the mean scores of acceptability range from 6 to 8 | If the mean scores of acceptability < 6 | Acceptability was assessed using a Likert scale from 0 (not at all acceptable) to 10 (completely acceptable); We classified the programme as "acceptable" if it received an average Likert scale score of 6 or higher, indicating a positive perspective beyond neutrality (typically defined as a score of 5). | Go-proceed with RCT |
| **Feasibility of measurements assessment**   1. Can 80% of participants complete all the measurements? 2. Can at least 80% of completed measurements with missing values < 10%? (Cases with missing values ≥ 10% for one or more time points will be removed from the database before performing repeated measures analysis of variance) | 1. If ≥ 80% of participants complete all the measurements   AND   1. If ≥ 80% of completed measurements with missing values < 10% | 1. If 70%-80% of participants complete all the measurements   AND   1. If ≥ 80% of completed measurements with missing values < 10% | 1. If < 70% of participants complete all the measurements   OR   1. If < 80% of completed measurements with missing values < 10% at each time point | 1. Several studies (3, 4) reported completion rates of measurements ranging from 83% (5) to 93.5% (8). This criterion is also in line with the dropout rate of *Feasibility of participant retention.* 2. Missing values > 10% is one of the criteria for exclusion from analysis. Some relevant trials reported 5.8%-22% exclusion due to missing or inconsistent data (4, 9). | Go-proceed with RCT |
| **Delivery of intervention content**  Can 80% of the intervention content be delivered as planned which is checked by the fidelity checklist based on audio recordings? | If ≥ 80% of intervention content is delivered | If 70%-80% of intervention content is delivered | If < 70% of intervention content is delivered | A fidelity checklist was developed following the previous evidence (10-12) (two researchers checked the audio recordings to see if the programme was delivered as planned). | Go-proceed with RCT |

**Table S4 Baseline characteristics of participants in two groups**

| **Variables** | **Intervention group**  ***n* = 40** | **Control group**  ***n* = 42** | ***T*** | ***P*** |
| --- | --- | --- | --- | --- |
| Child age (years) | 4.58 ± 0.92 | 4.78 ± 0.88 | -1.036 ^a^ | 0.303 |
| Child sex |  |  |  |  |
| Boys | 17 (42.5) | 22 (52.4) | 0.802^b^ | 0.370 |
| Girls | 23 (57.5) | 20 (47.6) |  |  |
| Child weight (BMI Z-score) |  |  |  |  |
| Underweight: BMI Z-score < -2 | 0 | 0 | 1.416 ^d^ | 0.546 |
| Normal weight: -2 ≤ BMI Z-score ≤ 1 | 29 (72.5) | 32 (76.2) |  |  |
| Overweight: 1 < BMI Z-score ≤ 2 | 5 (12.5) | 7 (16.7) |  |  |
| Obesity: BMI Z-score >2 | 6 (15.0) | 3 (7.1) |  |  |
| Duration of breastfeeding |  |  |  |  |
| 0-6 months | 6 (15.0) | 7 (16.7) | 0.402 ^b^ | 0.818 |
| 6-12 months | 22 (55.0) | 25 (59.5) |  |  |
| More than 12 months | 12 (30.0) | 10 (23.8) |  |  |
| The role of caregivers |  |  |  |  |
| Mothers | 38 (95.0) | 39 (92.9) |  |  |
| Fathers | 2 (5.0) | 3 (7.1) |  |  |
| Parental age | 33.73 ± 3.35 | 33.40 ± 3.43 | 0.422 ^a^ | 0.675 |
| Parental weight status |  |  |  |  |
| Underweight: BMI < 18.5 kg/m² | 4 (10.0) | 3 (7.1) | 0.550 ^d^ | 0.786 |
| Normal weight: 18.5 kg/m² ≤ BMI < 24.0 kg/m² | 26 (65.0) | 26 (61.9) |  |  |
| Overweight or Obesity: BMI ≥ 24.0 kg/m² | 10 (25.0) | 13 (31.0) |  |  |
| Parental education level |  |  |  |  |
| Senior High School or below | 21 (52.5) | 20 (47.6) | 1.797 ^b^ | 0.407 |
| Junior College | 14 (35.0) | 12 (28.6) |  |  |
| College or higher | 5 (12.5) | 10 (23.8) |  |  |
| Number of the children |  |  |  |  |
| One | 13 (32.5) | 15 (35.7) | 0.094 ^b^ | 0.759 |
| Two or more | 27 (67.5) | 27 (64.3) |  |  |
| Family structure |  |  |  |  |
| Co-parenting: grandparents and parents | 10 (25.0) | 19 (45.2) | 3.671 ^a^ | 0.055 |
| Only parents | 30 (75.0) | 23 (54.8) |  |  |
| Household income/year |  |  |  |  |
| Below average (≤ CNY 150,000) | 23 (57.5) | 24 (57.1) | 0.001 ^b^ | 0.974 |
| Above average (> CNY 150,000) | 17 (42.5) | 18 (42.9) |  |  |
| Concern about child overweight | 1.88 ± 1.042 | 1.86 ± 0.843 | 0.085 ^a^ | 0.932 |
| Concern about child overweight | 2.13 ± 1.067 | 2.45 ± 1.109 | -1.361 ^a^ | 0.177 |
| Self-reported perception of child weight |  |  |  |  |
| Underweight | 16 (40.0) | 11 (26.2) | 1.976 ^c^ | 0.372 |
| Normal weight | 19 (47.5) | 26 (61.9) |  |  |
| Overweight/obese | 5 (12.5) | 5 (11.9) |  |  |
| Visual perception of child weight |  |  |  |  |
| Thin | 14 (35.0) | 15 (35.4) | 0.156 ^b^ | 0.925 |
| Just right | 17 (42.5) | 19 (43.9) |  |  |
| Heavy | 9 (22.5) | 8 (20.7) |  |  |
| Accurate self-reported perception of child weight |  |  |  |  |
| Misperception | 21 (52.5) | 22 (52.4) | 0.000 ^b^ | 0.991 |
| Non-misperception | 19 (47.5) | 20 (47.6) |  |  |
| Accurate visual perception of child weight |  |  |  |  |
| Misperception | 19 (47.5) | 25 (59.5) | 1.191 ^b^ | 0.275 |
| Non-misperception | 21 (52.5) | 17 (40.5) |  |  |
| Encouragement of healthy eating | 4.18 ± 0.52 | 4.24 ± 0.50 | -0.560 ^a^ | 0.577 |
| Modelling | 3.98 ± 0.78 | 4.02 ± 0.97 | -0.197 ^a^ | 0.844 |
| Monitoring | 4.06 ± 0.75 | 4.19 ± 0.81 | -0.741 ^a^ | 0.461 |
| Pressure to eat | 3.18 ± 0.86 | 3.45 ± 0.85 | -1.464 ^a^ | 0.147 |
| Food as a reward | 3.45 ± 0.74 | 3.63 ± 0.89 | -0.998 ^a^ | 0.321 |
| Restriction | 3.49 ± 0.84 | 3.43 ± 0.97 | 0.294 ^a^ | 0.770 |
| Food fussiness | 2.99 ± 0.78 | 2.95 ± 0.74 | 0.253 ^a^ | 0.801 |
| Satiety responsiveness | 2.62 ± 0.74 | 2.59 ± 0.68 | 0.188 ^a^ | 0.851 |
| Food responsiveness | 2.58 ± 0.65 | 2.38 ± 0.61 | 1.401 ^a^ | 0.165 |
| Emotional eating | 1.67 ± 0.53 | 1.70 ± 0.54 | -0.256 ^a^ | 0.799 |
| Initiative eating | 4.00 ± 0.53 | 3.79 ± 0.60 | 1.714 ^a^ | 0.090 |
| PSOC: efficacy | 37.38 ± 4.82 | 36.90 ± 4.88 | 0.439 ^a^ | 0.662 |
| PSOC: satisfaction | 35.03 ± 8.75 | 36.21 ± 7.61 | -0.658 ^a^ | 0.513 |

*Notes.* Data are shown as n (%) or mean ± SD

^a^ Independent-Sample T-Test; ^b^ Pearson chi-square; ^c^ continuity correction; ^d^ fisher’s exact test

BMI: body mass index; PSOC: Parenting Sense of Competence Scale.

**Table S5. Fidelity assessment of the EPO-Feeding Programme**

| **Fidelity domain** | **Elements** | **Main references** | **Fidelity Score** | **Comments** |
| --- | --- | --- | --- | --- |
| Study design | - Treatment dose in the intervention and control groups - Provider credentials - Theoretical model - Potential barriers | - Protocol - Intervention manual | 9/10 | The study design adheres closely to the protocol and manual. |
| Training of Providers | - The training process - Training standards - Assessment - Education/working field | - Protocol - Scheduled meetings - Research background of providers | 9/10 | Muti-time meetings and discussions were conducted for training. Two providers were healthcare professionals who had over 10 years of working experience in child health in the local hospital. |
| Programme Delivery | - The delivery of each module - Methods to ensure the intervention content and dosage as specified - Adherence to the intervention plan - Plan to avoid contamination | - Auto recordings - Prepared materials - Observation/field notes of modules - Protocol - Intervention manual | Module 1: 9/10  Module 2: 8/10  Module 3: 9/10  Module 4: 9/10 | The programme was delivered as intended in the majority of cases. Some modules could benefit from improved interaction with participants. |
| Programme Receipt | - Assessment of understanding the intervention - Strategies to improve participants’ understanding - Participants’ engagement with intervention - Completion of homework activities | - Auto recordings - Interviews | 9/10 | Most participants expressed that they "did not have any difficulties" in understanding the content. Attendance rates for the modules and completion of homework activities were also high. The instructions for homework should be clearer. |
| Programme Enactment | - Application of knowledge and skills - Behavioural change | - Auto recordings - Intervention manual - Interviews | 8/10 | Many parents reported positive changes in their feeding practices, while only a few reported minimal changes due to external factors. |

**Table S6. Outcomes comparison for both groups compared to baseline (T_0_)**

|  | I | J | EPO-Feeding Programme + Usual care | | | Usual care | | |
| --- | --- | --- | --- | --- | --- | --- | --- | --- |
|  |  |  | Mean difference (I-J) | P value | 95% CI  (Lower, Upper) | Mean difference (I-J) | P value | 95% CI  (Lower, Upper) |
| Encouragement of healthy eating | T0 | T1 | -0.204* | 0.013 | (-0.373, -0.035) | 0.060 | 1.000 | (-0.138, 0.257) |
|  |  | T2 | -0.200 | 0.061 | (-0.407, 0.007) | 0.123 | 0.701 | (-0.131, 0.377) |
| Modelling | T0 | T1 | -0.354* | 0.010 | (-0.635, -0.072) | -0.024 | 1.000 | (-0.403, 0.356) |
|  |  | T2 | -0.200 | 0.346 | (-0.511, 0.111) | -0.065 | 1.000 | (-0.458, 0.329) |
| Monitoring | T0 | T1 | -0.125 | 0.554 | (-0.357, 0.107) | -0.030 | 1.000 | (-0.316, 0.257) |
|  |  | T2 | -0.350* | 0.010 | (-0.629, -0.071) | 0.149 | 0.692 | (-0.156, 0.454) |
| Pressure to eat | T0 | T1 | 0.333* | 0.042 | (0.009, 0.658) | 0.183 | 0.655 | (-0.182, 0.547) |
|  |  | T2 | 0.650*** | <.001 | (0.244, 1.056) | 0.167 | 0.492 | (-0.127, 0.460) |
| Food as a reward | T0 | T1 | 0.475** | 0.001 | (0.165, 0.785) | 0.071 | 1.000 | (-0.283, 0.426) |
|  |  | T2 | 0.650*** | <.001 | (0.333, 0.967) | 0.119 | 1.000 | (-0.261, 0.499) |
| Restriction | T0 | T1 | -0.306 | 0.065 | (-0.626, 0.014) | -0.196 | 0.339 | (-0.499, 0.106) |
|  |  | T2 | -0.100 | 1.000 | (-0.435, 0.235) | -0.304 | 0.195 | (-0.703, 0.096) |
| Food fussiness | T0 | T1 | 0.195 | 0.115 | (-0.033, 0.423) | -0.010 | 1.000 | (-0.249, 0.230) |
|  |  | T2 | 0.030 | 1.000 | (-0.162, 0.222) | 0.110 | 0.868 | (-0.145, 0.364) |
| Satiety responsiveness | T0 | T1 | 0.075 | 1.000 | (-0.136, 0.286) | 0.029 | 1.000 | (-0.124, 0.181) |
|  |  | T2 | 0.010 | 1.000 | (-0.185, 0.205) | -0.024 | 1.000 | (-0.182, 0.134) |
| Food responsiveness | T0 | T1 | 0.104 | 0.709 | (-0.113, 0.321) | -0.028 | 1.000 | (-0.242, 0.186) |
|  |  | T2 | 0.096 | 0.676 | (-0.099, 0.290) | -0.036 | 1.000 | (-0.225, 0.153) |
| Emotional eating | T0 | T1 | 0.020 | 1.000 | (-0.233, 0.273) | -0.105 | 0.691 | (-0.319, 0.110) |
|  |  | T2 | -0.030 | 1.000 | (-0.288, 0.228) | -0.195* | 0.034 | (-0.379, -0.012) |
| Initiative eating | T0 | T1 | 0.005 | 1.000 | (-0.144, 0.154) | 0.010 | 1.000 | (-0.165, 0.184) |
|  |  | T2 | 0.115 | 0.229 | (-0.043, 0.273) | -0.014 | 1.000 | (-0.203, 0.174) |
| PSOC: efficacy | T0 | T1 | -1.700 | 0.065 | (-3.476, 0.076) | -0.143 | 1.000 | (-2.106, 1.820) |
|  |  | T2 | -1.925* | 0.002 | (-3.237, -0.613) | 0.452 | 1.000 | (-1.275, 2.180) |
| PSOC: satisfaction | T0 | T1 | -0.975 | 0.748 | (-3.061, 1.111) | -1.429 | 0.362 | (-3.680, 0.822) |
|  |  | T2 | -2.325* | 0.016 | (-4.290, -0.360) | -0.500 | 1.000 | (-2.867, 1.867) |

*Note*. The statistical test used repeated measures ANOVA. CI: Confidence Interval

* *P* < 0.05, ** *P* < 0.01, *** *P* < 0.001

**Table S7. Generalised estimating equations results for the main effects and interaction effects**

| **Outcomes** | **Time effects** | | | **Group effects** | | | **Group*time effect** | | |
| --- | --- | --- | --- | --- | --- | --- | --- | --- | --- |
|  | **Wald χ2** | ***df*** | ***P*** | **Wald χ2** | ***df*** | ***P*** | **Wald χ2** | ***df*** | ***P*** |
| Accurate self-reported perception of child weight  (non-misperception vs. misperception) | 8.886* | 2 | 0.012 | 2.871 | 1 | 0.090 | 6.521* | 2 | 0.038 |
| Accurate visual perception of child weight  (non-misperception vs. misperception) | 7.315* | 2 | 0.026 | 9.170** | 1 | 0.002 | 4.188 | 2 | 0.123 |
| Actual child weight status | 3.728 | 2 | 0.155 | 0.024 | 1 | 0.877 | 5.569 | 2 | 0.062 |

*Note.* **P*＜0.05, ***P*＜0.01, ****P*＜0.001.

**Table S8. Results of Generalised estimating equation analysis for comparison of outcome variables between the intervention and control group**

| **Outcomes** | **Intervention group** | | **Control group** | | ***P_int_*** |
| --- | --- | --- | --- | --- | --- |
|  | **OR (95%CI)** | ***P*** | **OR (95%CI)** | ***P*** |  |
| **Accurate self-reported perception of child weight: non-misperception vs. misperception** |  |  |  |  |  |
| T_0_ (Baseline)  Misperception IG *n* = 21 (52.5%); CG *n* = 22 (52.4%)  Non-misperception IG *n* = 19 (47.5%); CG *n* = 20 (47.6%) |  |  |  |  |  |
| T_1_  Misperception IG *n* = 12 (30.0%); CG *n* = 20 (47.6%)  Non-misperception IG *n* = 28 (70.0%); CG *n* = 22 (52.4%) | 0.388 (0.220, 0.683) | 0.001 | 0.826 (0.411, 1.660) | 0.592 | 0.099 |
| T_2_  Misperception IG *n* = 10 (25.0%); CG *n* = 21 (50%)  Non-misperception IG *n* = 30 (75.0%); CG *n* = 21 (50%) | 0.302 (0.159, 0.572) | < 0.001 | 0.909 (.464, 1.782) | 0.781 | 0.020 |
| **Accurate visual perception of child weight:**  **non-misperception vs. misperception** |  |  |  |  |  |
| T_0_ (Baseline)  Misperception IG *n* = 19 (47.5%); CG *n* = 25 (59.5%)  Non-misperception IG *n* = 21 (52.5%); CG *n* = 17 (40.5%) |  |  |  |  |  |
| T_1_  Misperception IG *n* = 15 (37.5%); CG *n* = 26 (61.9%)  Non-misperception IG *n* = 25 (62.5%); CG *n* = 16 (38.1%) | 1.508 (1.549, 8.458) | 0.281 | 0.905 (0.473, 1.731) | 0.763 | 0.312 |
| T_2_  Misperception IG *n* = 8 (20.0%); CG *n* = 23 (54.8%)  Non-misperception IG *n* = 32 (80.0%); CG *n* = 19 (45.2%) | 3.619 (0.714, 3.184) | 0.003 | 1.215 (0.628, 2.348) | 0.563 | 0.046 |
| **Actual child weight status** |  |  |  |  |  |
| T_0_ (Baseline)  Normal weight IG *n* = 29 (72.5%); CG *n* = 32 (76.2%)  Overweight IG *n* = 5 (12.5%); CG *n* = 7 (16.7%)  Obesity IG *n* = 6 (15.0%); CG *n* = 3 (7.1%) |  |  |  |  |  |
| T_1_  Underweight IG *n* = 0 (0%); CG *n* = 1 (2.4%)  Normal weight IG *n* = 33 (82.5%); CG *n* = 28 (66.7%)  Overweight IG *n* = 1 (2.5%); CG *n* = 10 (23.8%)  Obesity IG *n* = 6 (15.0%); CG *n* = 3 (7.1%) | 0.926 (0.709, 1.209) | 0.524 | 1.145 (0.725, 1.808) | 0.560 | 0.255 |
| T_2_  Normal weight IG *n* = 27 (67.5%); CG *n* = 30 (71.4%)  Overweight IG *n* = 8 (20%); CG *n* = 8 (19.0%)  Obesity IG *n* = 5 (12.5%); CG *n* = 4 (9.5%) | 1.142 (0.759, 1.717) | 0.571 | 1.263 (0.737, 2.165) | 0.395 | 0.919 |

*Note.* OR: Odds Ratio; CI: Confidence Interval; *P*_int_: P value for interaction effect test; IG: intervention group; CG: Control group.

1. Whitehead AL, Julious SA, Cooper CL, Campbell MJ. Estimating the sample size for a pilot randomised trial to minimise the overall trial sample size for the external pilot and main trial for a continuous outcome variable. Stat Methods Med Res. 2016;25(3):1057-73.

2. Teresi JA, Yu X, Stewart AL, Hays RD. Guidelines for Designing and Evaluating Feasibility Pilot Studies. Med Care. 2022;60(1):95-103.

3. Sobko T, Brown GTL, Cheng WHG. Does connectedness to nature improve the eating behaviours of pre-schoolers? Emerging evidence from the Play&Grow randomised controlled trial in Hong Kong. Appetite. 2020;154:104781.

4. Haire-Joshu D, Elliott MB, Caito NM, Hessler K, Nanney MS, Hale N, et al. High 5 for Kids: the impact of a home visiting program on fruit and vegetable intake of parents and their preschool children. Prev Med. 2008;47(1):77-82.

5. Hart LM, Damiano SR, Paxton SJ. Confident body, confident child: A randomized controlled trial evaluation of a parenting resource for promoting healthy body image and eating patterns in 2- to 6-year old children. Int J Eat Disord. 2016;49(5):458-72.

6. Hammersley ML, Okely AD, Batterham MJ, Jones RA. An Internet-Based Childhood Obesity Prevention Program (Time2bHealthy) for Parents of Preschool-Aged Children: Randomized Controlled Trial. J Med Internet Res. 2019;21(2):e11964.

7. Gomes AI, Barros L, Pereira AI, Roberto MS. Effectiveness of a parental school-based intervention to improve young children's eating patterns: a pilot study. Public Health Nutr. 2018;21(13):2485-96.

8. Agras WS, Hammer LD, Huffman LC, Mascola A, Bryson SW, Danaher C. Improving healthy eating in families with a toddler at risk for overweight: a cluster randomized controlled trial. J Dev Behav Pediatr. 2012;33(7):529-34.

9. Morshed AB, Tabak RG, Schwarz CD, Haire-Joshu D. The Impact of a Healthy Weight Intervention Embedded in a Home-Visiting Program on Children's Weight and Mothers' Feeding Practices. J Nutr Educ Behav. 2019;51(2):237-44.

10. Borrelli B. The assessment, monitoring, and enhancement of treatment fidelity in public health clinical trials. J Public Health Dent. 2011;71 Suppl 1:S52-63.

11. Toomey E, Matvienko-Sikar K, Heary C, Delaney L, Queally M, Hayes CB, et al. Intervention Fidelity Within Trials of Infant Feeding Behavioral Interventions to Prevent Childhood Obesity: A Systematic Review. Ann Behav Med. 2019;53(1):75-97.

12. Borrelli B, Sepinwall D, Ernst D, Bellg AJ, Czajkowski S, Breger R, et al. A new tool to assess treatment fidelity and evaluation of treatment fidelity across 10 years of health behavior research. J Consult Clin Psychol. 2005;73(5):852-60.
